# Isopentenyltransferases as master regulators of crop performance: their function, manipulation, and genetic potential for stress adaptation and yield improvement

**DOI:** 10.1111/pbi.13603

**Published:** 2021-05-02

**Authors:** Hai Ngoc Nguyen, Nhan Lai, Anna B. Kisiala, R. J. Neil Emery

**Affiliations:** ^1^ Department of Biology Trent University Peterborough ON Canada; ^2^ School of Biotechnology Vietnam National University Ho Chi Minh City Vietnam

**Keywords:** *IPT*, plant yield, cytokinin, phytohormone, abiotic stress, biotic stress, stress response

## Abstract

Isopentenyltransferase (IPT) in plants regulates a rate‐limiting step of cytokinin (CTK) biosynthesis. *IPTs* are recognized as key regulators of CTK homeostasis and phytohormone crosstalk in both biotic and abiotic stress responses. Recent research has revealed the regulatory function of *IPTs* in gene expression and metabolite profiles including source‐sink modifications, energy metabolism, nutrient allocation and storage, stress defence and signalling pathways, protein synthesis and transport, and membrane transport. This suggests that *IPT*s play a crucial role in plant growth and adaptation. *In planta* studies of *IPT*‐driven modifications indicate that, at a physiological level, *IPTs* improve stay‐green characteristics, delay senescence, reduce stress‐induced oxidative damage and protect photosynthetic machinery. Subsequently, these improvements often manifest as enhanced or stabilized crop yields and this is especially apparent under environmental stress. These mechanisms merit consideration of the *IPTs* as ‘master regulators’ of core cellular metabolic pathways, thus adjusting plant homeostasis/adaptive responses to altered environmental stresses, to maximize yield potential. If their expression can be adequately controlled, both spatially and temporally, *IPT*s can be a key driver for seed yield. In this review, we give a comprehensive overview of recent findings on how *IPT*s influence plant stress physiology and yield, and we highlight areas for future research.

## Introduction

### Multiple functions of CTKs in plant development and adaptation

Biotic and abiotic stresses negatively affect plant growth and development. The abiotic stresses which are major constraints in crop production include temperature stress (heat and cold), osmotic stress (drought and salinity), heavy metal exposure, irradiation, and oxidative stress. On the biotic side, attacks by pathogenic fungi, bacteria, viruses, and oomycetes are major stress factors, which impose losses in global agricultural production. During evolution, plants have developed a series of complex mechanisms to deal with environmental stressors. The communication from environmental sensing to an effective response is regulated by phytohormones. Cytokinins (CTKs) are adenine derivatives with isoprenoid or aromatic side chains; they represent a major phytohormone group and several of them act as key regulators for plant development and stress adaptation (Li *et al.,* 2019; Wybouw and De Rybel, 2019). Cytokinins play pivotal roles in apical dominance, shoot meristem function, root expansion, source‐sink relationships, leaf senescence, reproductive development, and seed filling. Cytokinins are thus involved in many qualitative and quantitative components of yield. These regulatory functions make CTKs fascinating signalling molecules and there is much incentive to unravel their biosynthesis and signalling pathways in plants (Kieber and Schaller, 2014; Wybouw and De Rybel, 2019). Cytokinin action, induced by environmental stimuli, often invokes hormonal crosstalk. This, in turn, orchestrates the synthesis, transport, and function of other plant growth regulators, under both abiotic and biotic stresses. It is suggested that CTKs have evolved as a bridge in coordinating endogenous developmental processes and adaptive responses (Li *et al.,* 2016, 2019).

**Figure 1:**
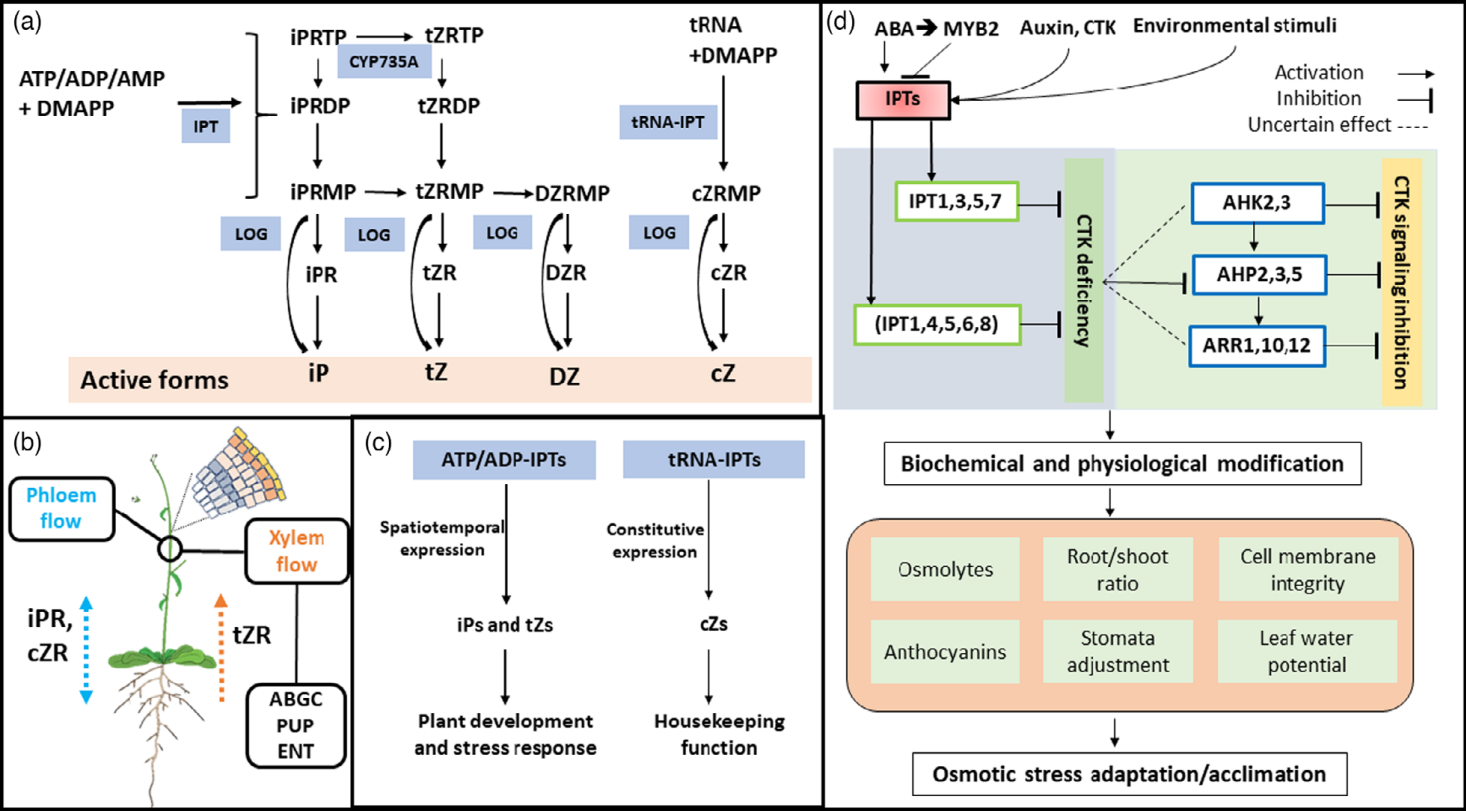
An overview of CTK biosynthesis (a); CTK translocation (b); general characteristics of two known CTKs biosynthesis pathways in angiosperms (c) and, the mode of action of *IPTs* in drought tolerance in Arabidopsis (d). (a) CTK types derived from two known pathways, adenylate (*ATP/ADP‐IPTs* and *AMP‐IPTs*) and tRNA types (adapted from Hirose *et al*. (2008), further details in Sakakibara (2006)). ADP: adenosine 5’‐diphosphate; ATP: adenosine 5’‐triphosphate; tRNA: Transfer ribonucleic acid; DMAPP: dimethylallyl diphosphate; IPT: isopentenyl transferase; CYP735A: cytochrome P450 monooxygenase, family 735, subfamily A (cytokinin *trans‐*hydroxylase); LOG: cytokinin phosphoribohydrolase ‘Lonely guy’; *c*Z: *cis*‐Zeatin, *c*ZR: *c*Z riboside; *c*ZRMP: *c*Z riboside 5’‐monophosphate, DZ: Dihydrozeatin, DZR: DZ riboside; DZRMP: DZ riboside 5’‐monophosphate; iP: isopentenyladenine; iPR: iP riboside; iPRMP: isopentenyladenosine‐5’–monophosphate; iPRDP: isopentenyladenosine‐5'‐diphosphate; iPRTP: isopentenyladenosine‐5'‐triphosphate; *t*Z: *trans*‐Zeatin, *t*ZR: *t*Z riboside, *t*ZRDP: *t*Z riboside 5’‐diphosphate; *t*ZRMP: *t*Z riboside 5’‐monophosphate; *t*ZRTP: *t*Z riboside 5’‐triphosphate. (b) Translocation of CTKs in plant. As multifunctional and mobile signalling molecules, active CTKs and their derivatives contribute to various developmental processes depending on CTK transport proteins across vascular tissues. Briefly, three kinds of CTK transporters have been systematically characterized: equilibrate nucleoside transporters (ENT), purine permeases (PUP), and G subfamily ATP‐binding cassette (ABCG) transporters (Durán‐Medina *et al.,* 2017). *t*ZR is the primary form of xylem transported CTKs, and iPR and *c*ZR are major forms of phloem CTKs (Osugi and Sakakibara, 2015). Arabidopsis plant vector is adapted, with permission, from Figshare (Bouché, 2018). (c) General mode of action of two *IPT* GFMs (*ATP/ADP‐IPTs and tRNA‐IPTs*) in angiosperms (Köllmer et al 2014; Miyawaki et al 2006; Wang *et al.,* 2020a). The investigation in Arabidopsis mutants showed that *ATP/ADP‐IPT* GFMs control biosynthesis of iP‐ and *t*Z‐type CTKs while tRNA‐type *IPT* genes regulate *c*Z‐type CTKs (Köllmer *et al.,* 2014; Miyawaki *et al.,* 2006). In the basal angiosperm *Amborella* *trichopoda (Amborellaceae)*, and in *Fragaria vesca* (wild strawberry), eudicot woodland strawberry, transcriptomic analysis indicated that *tRNA‐IPTs* are constitutively expressed throughout the plant, whereas the expression of *ATP/ADP‐IPTs* is tissue‐specific and rapidly down‐regulated by abiotic stresses (Wang *et al.,* 2020a). Generally, *tRNA‐IPTs* and the associated *c*Z‐type CTKs play a housekeeping role, whereas *ATP/ADP‐IPTs* and associated iP/*t*Z‐type CTKs play regulatory roles in organ development and stress responses in angiosperms (Köllmer *et al.,* 2014; Miyawaki *et al.,* 2004, 2006; Wang *et al.,* 2020a). (d) The negative regulatory role of *IPT*‐repressed CTKs in osmotic stress tolerance in Arabidopsis. Arrowheads represent activation, and perpendicular bars indicate inhibition. Studies of drought stress tolerance using CTK‐deficient plants, such as the quadruple *ATP/ADP‐ipt1,3,5,7* loss‐of‐function mutants, CTK signalling mutants [*AHP* mutants (Arabidopsis Histidine Phosphotransfer Proteins 2,3,5) and *ARR* mutants (type B Arabidopsis Response Regulators 1,10,12)], and down‐regulated expression of five *ATP/ADP‐IPT* genes (*IPT1, IPT4, IPT5, IPT6, IPT8*) found in the *AtMYB2* (ABA‐dependent signalling pathway) overexpressor, have indicated that CTK‐depleted mutants have improved drought acclimation/adaptation (Guo and Gan, 2011; Nguyen *et al.,* 2016; Nishiyama *et al.,* 2011, 2013; Werner *et al.,* 2010). Briefly, reducing endogenous CTK lowers the output of the CTK signalling cascades (i.e. quadruple *ipt1,3,5,7* down‐regulates expression of *AHP2*, *AHP3*, *AHP5*) leading to drought acclimation/adaptation. CTK: cytokinin; AHK: Arabidopsis histidine kinase; AHP: Arabidopsis histidine phosphotransfer; ARR: Arabidopsis response regulator; ROS: reactive oxygen species.

Plant CTK homeostasis is complex and depends mainly on a number of key pathway enzymes in: biosynthesis [adenosine phosphate‐isopentenyltransferase (ATP/ADP‐IPTs) and tRNA‐isopentenyltransferase (tRNA‐IPT)]; side‐chain modification [cytokinin trans‐hydroxylase (CYP735A)]; O‐glucosylation [zeatin O‐glucosyltransferase (ZOG)]; hydrolysis of *O*‐glucosides [β‐glucosidase (GLU)]; N‐glucosylation [UDP‐glycosyltransferase (UGT)] primary activation [LONELY GUY (LOG)]; and degradation [cytokinin oxidase/dehydrogenase (CKX)] (Sakakibara, 2006). Cytokinins are known to occur in plants in at least 32 different forms (Kisiala *et al.,* 2019). They are distributed and translocated among plant organs, and affect multiple biological processes, by way of their varying chemical properties and metabolic and signalling mechanisms (Durán‐Medina *et al.,* 2017). An overview of CTK synthesis and translocation is summarized in Figure 1a and b. The initial steps in plant CTK biosynthesis are regulated by *IPT* gene family members (GFMs), which can be classified either into the adenylate *ATP/ADP‐IPT* GFMs or *tRNA‐IPT* GFMs (Kieber and Schaller, 2018). Generally, the *ATP/ADP‐IPTs* account for the biosynthesis of isopentenyladenine (iP)‐ and *trans*‐zeatin (*t*Z)‐type CTKs via using ATP or ADP as their preferred substrates, whereas *tRNA‐IPTs* are responsible for the synthesis of *cis*‐zeatin (*c*Z)‐type CTKs via transferring isopentenyl groups to the N^6^ atom of adenines in tRNAs (Figure 1a) (Sakakibara, 2006).

Genome‐wide association studies for identification and annotation of novel *IPT* GFMs as well as altering the expression of *IPTs* have provided a new strategy for engineering CTKs for contemporary plant breeding programmes (Jameson and Song, 2016; Kieber and Schaller, 2018). For example, multiple Arabidopsis studies have indicated that manipulation of CTK levels via *IPTs* can enhance drought and salt stress tolerance (Li *et al.,* 2016, 2019). Plants with modified expression of *IPTs* show improved growth and enhanced yield, opening new areas of exploration for future crop improvement technologies (Jameson and Song, 2016). Here, we critically review the current knowledge about *IPTs*, including their origins, evolution, induced‐stress sensing, and their roles in stress response and tolerance. We also discuss recent developments in elucidating the signalling pathways for plant stress physiology and field‐based achievements through *in planta* studies of *IPTs*. This review focuses on how *IPTs* are activated and connected to upstream sensing mechanisms and to downstream molecular cascades, which is attributed to changes in hormonal crosstalk, altered cellular metabolism, plant physiological responses, and plant yield.

### A portrait of IPTs in the plant kingdom

There are three characteristics of plant *IPT* genes which point to their crucial role in plant development, yield formation, and adaptation processes: (i) *IPTs* are widespread in the plant kingdom; (ii) *IPT* expression occurs spatially throughout plant anatomy and temporally during all plant developmental stages; (iii) *IPTs* actively respond to environmental stresses including both abiotic and biotic factors, and to other phytohormones. These features make *IPTs* key developmental and adaptive regulators during plant evolution.

#### IPTs – ancient signalling molecules

For a long time, *IPTs* had been known to exist in various groups of bacteria and eukaryotes but not in Archaea (Lindner *et al.,* 2014; Nishii *et al.,* 2018). However, recently, putative *IPT* domains have been found in two archaeal species (belonging to the TACK superphylum), which have been proposed to be the direct ancestors of eukaryotes, suggesting that *IPT* homologs are also present in Archaea (Wang *et al.,* 2020a). Thus, *IPTs* exist as pan‐kingdom signalling molecules and can be found in bacteria, slime moulds, animals, fungi, SAR, algae, prasinophytes, core chlorophytes, charophytes, liverworts, mosses, hornworts, lycophytes, monilophytes, gymnosperms, and angiosperms (Nishii *et al.,* 2018).

Important efforts have been made to understand the complex history, origin, and classification of *IPTs* among kingdoms and within angiosperms. Frébort *et al*. (2011) classified *IPT* genes into five groups based on an unrooted gene tree reconstructed from full sequence lengths of *IPT* GFMs of two plant species, Arabidopsis and rice. To infer the deep origin and evolution of *IPTs* based on the matrix of a wide range of species, Lindner *et al*. (2014) performed a more comprehensive analysis using 30 species across different kingdoms. Nishii *et al*. (2018) focused their research on the conserved protein domain of the *IPT*s and found a deep history of *IPTs* that involved an interplay of possible horizontal gene transfers, gene duplication and loss events and functional diversification of *IPT* GFMs during evolution. Furthermore, through combined evolution and gene expression analyses of *tRNA‐IPTs* and *ATP/ADP‐IPTs* in land plants, it was determined that *tRNA‐IPTs* in angiosperms are associated with an initial endosymbiotic event, while *ATP/ADP‐IPTs* are derived from *tRNA‐IPTs*. Based on the combined unique transcriptional activities of these two known CTK biosynthesis pathways, together with knowledge from Arabidopsis mutant studies, a hypothesis was formed that *tRNA‐IPT* genes and the associated *c*Z‐type CTKs mainly play housekeeping roles, while ATP/ADP‐IPT genes and the associated iP/*t*Z‐type CTKs may be involved in regulating organ development and responses to environmental stresses (Köllmer *et al.,* 2014; Miyawaki *et al.,* 2004, 2006; Wang *et al.,* 2020a) (Figure 1c). However, *c*Zs are highly abundant in many plants (Schäfer *et al.,* 2015), and they are the predominant CTKs in sweet potato (Hashizume *et al.,* 1982), rice (Takagi *et al.,* 1985), potato (Nicander *et al.,* 1995), chickpea (Emery *et al.,* 1998), maize (Veach *et al.,* 2003), pea (Quesnelle and Emery, 2007) and moss (Lindner *et al.,* 2014). They are also the predominant CTKs, at certain developmental stages and in response to environmental cues (Schäfer *et al.,* 2015). A function of cZ in the regulation of plant response to abiotic stress, pathogen, and herbivore resistance has also been highlighted (Schäfer *et al.,* 2015). *cis*‐Zeatin might also play a role as a competitor to the more active CTKs, since they bind and activate CTK receptors in the moss, *P. patens* (Gruhn *et al.,* 2014), Arabidopsis (Spíchal *et al.,* 2004), and a wide range of crops such as maize (Yonekura‐Sakakibara *et al.,* 2004), rice (Choi *et al.,* 2012), or apple (Daudu *et al.,* 2017). However, in Arabidopsis, the trans‐isomers are by far the predominant forms at most growth stages (Gajdošová *et al.,* 2011) and their CTK receptors have a lower affinity with *c*Zs (Stolz *et al.,* 2011). The situation is different for CHASE‐domain‐containing histidine kinases (CHKs) from crop plants, like rice or apple, for which the differences in binding affinities among *t*Z, iP, and *c*Z do not show the same clear bias against *c*Z (Choi *et al.,* 2012; Daudu *et al.,* 2017). Therefore, the functional predictions based on previous investigations of Arabidopsis (a low *c*Z‐containing plant), and studies on the evolution and gene expression of *IPT* GFMs, probably do not reflect the role of *c*Z‐type CTKs *in planta* (Schäfer *et al.,* 2015). Many more experimental studies of *tRNA‐IPT* genes and the associated *c*Z‐type CTKs are needed.

#### IPTs in planta

**Figure 2:**
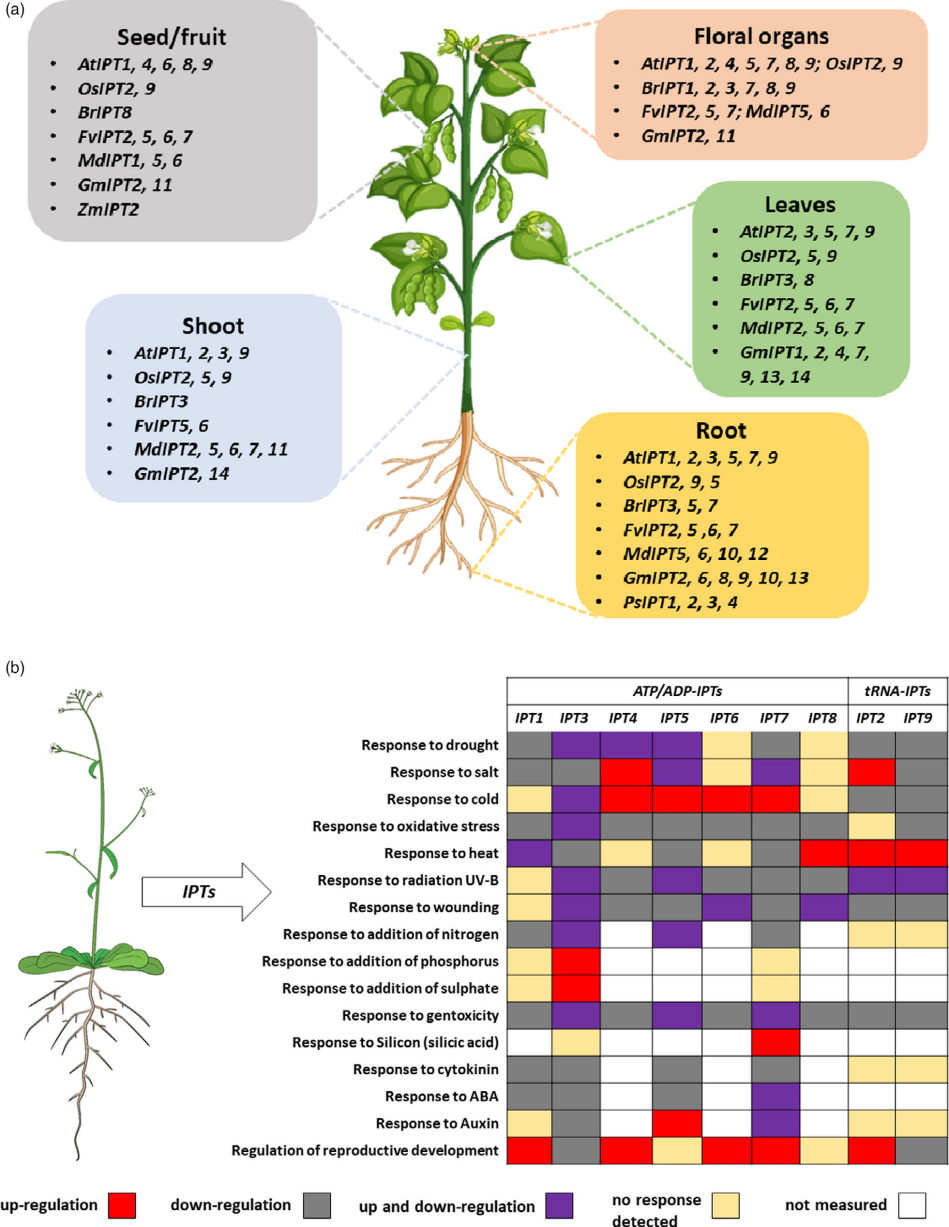
(a) Site‐specific expression of *IPT* GFMs in developmental tissues *in planta*, including Arabidopsis, rice, Chinese cabbage, strawberry, apple, soybean, pea, maize (Brugière *et al.,* 2008; Dolgikh *et al.,* 2017; Le *et al.,* 2012; Liu *et al.,* 2013b; Mi *et al.,* 2017; Miyawaki *et al.,* 2006; Takei *et al.,* 2001; Tan *et al.,* 2018; Tsai *et al.,* 2012). The names of the polyploid *BrIPT* GFMs follow the Liu *et al*. (2013b) naming system. (b) *IPT* GFMs in Arabidopsis are involved in a variety of environmental responses and developmental processes (Ghosh *et al.,* 2018; Hirose *et al.,* 2008; Markovich *et al.,* 2017; Miyawaki *et al.,* 2004; Nishiyama *et al.,* 2011; Takei *et al.,* 2002; Woo *et al.,* 2012). Color boxes indicate distinct transcriptional profiles of each *IPT* GFM in response to the noted stress factors or biological process Figure design is adapted from (Hallmark and Rashotte, 2019; Victor *et al.,* 2019). The Arabidopsis plant vector is adapted, with permission, from Figshare (Bouché, 2018).

*Isopentenyltransferase* GFMs are key enzymes in CTK biosynthesis, and they have been identified in a wide range of plant species including: nine *IPT* GFMs in Arabidopsis (Miyawaki *et al.,* 2006; Takei *et al.,* 2001); seven GFMs in hop (Sakano *et al.,* 2004); nine GFMs in maize (Brugière *et al.,* 2008); 10 GFMs in rice (Tsai *et al.,* 2012); 17 GFMs in soybean (Le *et al.,* 2012; Mens *et al.,* 2018); 13 GFMs in Chinese cabbage (Liu *et al.,* 2013b); six GFMs in pea (Dolgikh *et al.,* 2017); seven GFMs in strawberry (Mi *et al.,* 2017); 12 GFMs in apple (Tan *et al.,* 2018); and nine GFMs in kiwifruit (Nardozza *et al.,* 2020). In addition to identification of the *IPT* GFMs in various species, a systematic annotation of these putative genes has been conducted including characterization of their chromosome locus, enzyme domain features, and cis‐acting elements, among others. The diverse existence and unique expression of the *IPT* GFMs in crops emphasize the possibility for introducing *IPT* within or from one species to another to alter endogenous CTK levels for targeting desirable phenotypes (Figure 2). Upon exposure to the environmental changes, stress‐induced fluctuations in gene components of CTK metabolism can be associated with CTK action, including changes in *IPT* expression. Thus, it is important to understand *IPT* gene expression as an initial step to identifying stress response mechanisms and to develop new biotechnological strategies for enhancing plant performance under stress (Figure 2, Table 1).

**Table 1:** Impact of *IPT* genes on plant stress response and yield attributes. (↓) decrease, (↑) increase. Except for the loss of function study in Arabidopsis, all ectopic expressions of *IPT* genes were achieved using transformation with the *Agrobacterium tumefaciens IPT* gene

Plant species	Genetic engineering approach	Promoter control	Physiological alteration	Stress tolerance	Plant component impacted	Reference
Rice *(Oryza sativa)*	stress/maturation‐induced overexpression	*SARK*	↑ sink strength, photosynthesis; ↑ drought tolerance	Drought	↑ grain yield	Peleg *et al*. (2011)
Peanut (*Arachis hypogaea)*	stress/maturation‐induced overexpression	*SARK*	↑ photosynthesis; ↑ drought tolerance	Drought	↑ grain yield	Qin *et al*. (2011)
Canola *(Brassica napus)*	maturation‐induced overexpression	*AtMYB32*	↑ chlorophyll; ↓ senescence;	Drought	↑ grain yield	Kant *et al*. (2015)
Cotton *(Gossypium hirsutum)*	stress/maturation‐induced overexpression	*SARK*	↓ senescence; ↑ biomass; ↑ photosynthesis	Drought	↓ stress penalty	Kuppu *et al*. (2013)
	stress/senescence‐induced overexpression	*SAG12*	↓ senescence; ↑ drought response genes	Salt	↓ stress penalty	Liu *et al*. (2012); Zhao *et al*. (2013); Shan *et al*. (2019)
Creeping bentgrass *(Agrostis stolonifera)*	stress/senescence‐induced overexpression	*SAG12*	↑ ROS‐scavenging systems; ↑ metabolites; ↑ drought response genes	Drought	↓ stress penalty	Merewitz *et al*. (2010, 2012, 2016); Xu *et al*. (2016); Xu and Huang (2017)
		*HSP18.2*	↑ ROS‐scavenging systems; ↑ metabolites; ↑ drought response genes	Drought	↓ stress penalty	Merewitz *et al*. (2010, 2012, 2016); Xu *et al*. (2016); Xu and Huang (2017)
Wheat *(Triticum aestivum)*	maturation‐induced overexpression	Modified *AtMYB32*		Drought	↑ grain yield	Joshi *et al*. (2019)
Sweet potato *(Ipomoea batatas)*	stress/maturation‐induced overexpression	*SARK*	↓ senescence; ↑ photosynthesis; ↑ growth	Drought	↑ yield	Nawiri *et al*. (2018)
Tobacco *(Nicotiana tabacum)*	stress/maturation‐induced overexpression	*SARK*	↑ ABA and photosynthesis gene expression	Drought	↓ stress penalty	Rivero *et al*. (2010)
Eggplant (*Solanum melongena)*	stress/senescence‐induced overexpression	*SAG12*	↑ ROS‐scavenging systems; ↓ senescence	Drought	↓ stress penalty	Xiao *et al*. (2017)
Maize *(Zea mays)*	stress/maturation‐induced overexpression	*SARK*	↑ relative water content; ↑ chlorophyll	Drought	↑ grain yield	Bedada *et al*. (2016)
Broccoli *(Brassica oleracea var. italica)*	stress/senescence‐induced overexpression	*SAG*	↓ post‐harvest senescence		↓ senescence penalty	Liu *et al*. (2013a)
Arabidopsis *(Arabidopsis thaliana)*	stress/senescence‐induced overexpression	*SAG12*	↑ ABA, SA, ET	Drought	↓ stress penalty	Nishiyama *et al*. (2011)
	loss of function (*ipt1,3,5,7*)		↑ cell membrane integrity; ↑ ROS‐scavenging systems; ↑ drought response genes and metal‐binding encoded genes	Drought, salt, Selenium stress	↓ stress penalty	Nishiyama *et al*. (2012); Jiang *et al*. (2019)
Tobacco (*Nicotiana tabacum*)	Dexamethasone‐induced overexpression	*pOp/LhGR*	↑ necrotic lessons on leaves that prevent pathogen expansion	Biotic stress (pathogen attack)	↓ biomass penalty	Novák *et al*. (2013)
Arabidopsis (*Arabidopsis thaliana*)	stress‐induced overexpression	*35S*	↑ callose deposition upon *P. syringae* pv. *tomato* DC3000 infection	Biotic stress (pathogen attack)	↑ plant immunity	Choi *et al*. (2010)

#### Spatiotemporal expression of IPTs

In general, the *IPT* genes are expressed widely and not restricted to specific vegetative or reproductive organs (Figure 2a). However, the expression patterns of the adenylate *IPT*s show more tissue‐ and stage‐specificity compared with the more ubiquitous *tRNA‐IPTs* (Ghosh *et al.,* 2018; Liu *et al.,* 2013b; Miyawaki *et al.,* 2004). Arabidopsis has seven genes (*AtIPT1* and *AtIPT3–AtIPT8*) that encode *ATP/ADP‐IPTs* while two others (*AtIPT2* and *AtIPT9*) encode *tRNA‐IPTs*. Two Arabidopsis *tRNA‐IPTs* were ubiquitously expressed and showed the highest transcript levels within proliferating tissues (Ghosh *et al.,* 2018; Miyawaki *et al.,* 2004) while other *AtIPTs* exhibited specific, predominant expression patterns which varied among vegetative tissues such as phloem (*AtIPT3*), root primordia/cap (*AtIPT5*), siliques (*AtIPT6*), and other reproductive tissues such as immature seeds (*AtIPT1, 4, 8*), the chalazal endosperm (*AtIPT4, 8*) or pollen tubes (*AtIPT7*) (Miyawaki *et al.,* 2004). Any *IPT* GFMs that specifically contribute to vegetative organs (root, shoot, and leaves) and reproductive organs (seeds/fruits) in plants can be strategically targeted candidates for crop development through molecular and genetic techniques (Figure 2a).

#### The involvement of IPTs in plant stress responses

*Isopentenyltransferase* expression is known to strongly respond to either abiotic or biotic stresses (Ghosh *et al.,* 2018; Liu *et al.,* 2013b). Endogenous CTK levels, associated with the reduced *IPT* expression, generally decrease during exposure to abiotic stress, depending on stress duration and intensity (Alvarez *et al.,* 2008; Argueso *et al.,* 2009; Hare *et al.,* 1997), and this is opposite to trends observed for biotic stress. For example, CTK levels in infected plants often increase under infection with bacteria (Pertry *et al.,* 2009; Radhika *et al.,* 2015), fungi (Behr *et al.,* 2012; Jiang *et al.,* 2013; Morrison *et al.,* 2017), or insects (Brütting *et al.,* 2018; Schäfer *et al.,* 2015). However, the increase in CTK levels in infected plants most likely originates from the invader (Brütting *et al.,* 2018; Pertry *et al.,* 2009; Trdá *et al.,* 2017) and is not associated with any enhanced *IPT* expression.

In Arabidopsis, except for *AtIPT5*, gene expression of *AtIPT1, AtIPT3, and AtIPT7* was repressed during dehydration treatment. *AtIPT1* and *AtIPT3* were repressed during salt stress. *AtIPT5* and *AtIPT7* were slightly induced after 1 and 2h of NaCl treatment and their expression returned to basal levels after 5h of treatment (Nishiyama *et al.,* 2011). Transcriptional trajectories of all *IPT* GFMs in Arabidopsis, summarized in Figure 2b, have been thoroughly described before (Ghosh *et al.,* 2018; Hirose *et al.,* 2008; Markovich *et al.,* 2017; Miyawaki *et al.,* 2004; Nishiyama *et al.,* 2011; Takei *et al.,* 2002; Woo *et al.,* 2012). These data suggest that *IPTs* not only coordinate developmental processes under favourable growth conditions but are also important regulators of plant stress response. Furthermore, *IPTs* can be either negative or positive regulators of growth processes under stress exposure. For example, in rice, salt stress up‐regulated only *OsIPT5* levels and suppressed all other *OsIPT*s (Ghosh *et al.,* 2018). *OsIPT5* was up‐regulated in response to a range of other abiotic stresses and many toxic metals such as chromium, arsenic, lead, and cadmium. By contrast, *OsIPT5* was down‐regulated in response to multiple pathogenic treatments, including attacks by an insect (*Nilaparvata lugens*), nematodes (*Meloidogyne incognita* and *Meloidogyne graminicola*), a fungus (*Magnaporthe oryzae*), and a parasitic plant (*Striga hermonthica*) (Ghosh *et al.,* 2018). In soybean, CTK levels tend to decrease in leaves under drought conditions (Le *et al.,* 2012). Among the soybean *IPT* GFMs, *GmIPT08* and *GmIPT10* were up‐regulated in response to drought conditions in the reproductive stage (Le *et al.,* 2012). In Chinese cabbage, the majority of *BrIPT* GFMs were initially up‐regulated, before falling to basal levels during the extension of drought exposure, except for *BrIPT7*, for which transcription was continuously increased and maintained at high levels under severe drought conditions (Liu *et al.,* 2013b).

As CTK biosynthesis genes, *IPTs* play important roles also in response to the other phytohormones involved in plant stress adaptation. The influence of auxin and ABA on *IPT* transcriptional activities are depicted in Figure 2b. Exogenous ABA showed an inhibitory effect on CTK levels by suppressing *IPT* genes (Nishiyama *et al.,* 2011). The suppressed transcription of *AtIPT1*, *AtIPT3*, *AtIPT5* (but not *AtIPT7*) under ABA treatment suggests that excess ABA inhibits CTK biosynthesis (Nishiyama *et al.,* 2011). Besides ABA, treatment with other stress hormones, like jasmonic acid (JA) and salicylic acid (SA), could alter transcription of *IPT* genes. For example, in rice, *OsIPT2* and *OsIPT9* were down‐regulated by both JA and SA while *OsIPT5* expression was induced by these two hormones (Ghosh *et al.,* 2018). The occurrence of cis‐regulatory elements in *IPT* promoter regions also suggests there is involvement of *IPTs* in hormonal crosstalk. In particular, the analysis of *OsIPT* and *AtIPT* promoter sequences showed the presence of a variety of cis‐regulatory elements, including phytohormone‐sensitive, abiotic/biotic stress response, and development‐regulatory motifs and elements (Ghosh *et al.,* 2018). The *IPT* gene expression can thus provide a foundation for in‐depth characterization, and development of CTK‐based selective markers for crop breeding programmes focused on high yield crops via manipulation of CTK levels.

As discussed above, *IPTs* are developmental and adaptive communicators, and recent efforts have mainly focused on plant functional studies to validate the role of *IPTs* and clarify their expression patterns at molecular, cellular, and physiological levels. In the next section, we map out how *IPTs* precisely modulate plant tolerance under environmental stress, thereby mitigating losses and stabilizing crop yields.

## A path towards better‐engineered IPTs

### IPTs in plant abiotic stress physiology

In Arabidopisis, mutations in two *tRNA‐IPT* genes (*AtIPT2* and *AtIPT9*) do not affect the levels of iPs and *t*Zs but they significantly reduce levels of *c*Z‐type CTKs, resulting in plant chlorosis (Miyawaki *et al.,* 2006). CTK‐deficient plants with mutant *ATP/ADP‐IPTs* had reduced levels of iP‐type and *t*Z‐type CTKs but only slightly increased *c*Z content (Köllmer *et al.,* 2014). A quadruple *ATP/ADP‐IPT* (*ipt1,3,5,7*) Arabidopsis mutant with very low levels of iPs and *t*Zs, but slightly increased levels of *c*Zs, displayed significantly enhanced salt and drought tolerance (Li *et al.,* 2016; Nishiyama *et al.,* 2011). Research in Arabidopsis suggests that the ABA‐*AtMYB2* transcriptional factor (from the ABA‐dependent response pathway), down‐regulates several *ATP/ADP‐IPT* genes (*IPT1, IPT4, IPT5, IPT6,* and *IPT8*) leading to a CTK‐deficient phenotype and enhanced drought tolerance (Guo and Gan, 2011). The diverse physiological responses underlying increased abiotic stress tolerance in the CTK‐deficient Arabidopsis plants are listed in Figure 1d. These studies show that *ATP/ADP‐IPT*s and the associated iP/*t*Z‐type CTKs are negative regulators in plant responses to abiotic stress in Arabidopsis. The reduction in CTK content by quadruple loss of function of *ipt1,3,5,7* reduces the action of CTK signalling components (e.g. *AHP2*, *AHP3*, *AHP5*) which results in improved stress response and acclimation (Cortleven *et al.,* 2019; Li *et al.,* 2016; Nishiyama *et al.,* 2013). Suppression of CTK signalling (e.g. *AHP2, AHP3, AHP5, ARR1, ARR10, ARR12*) can lead to down‐regulation of many stress‐ and/or ABA‐responsive genes, and subsequently to drought‐tolerant phenotypes which exhibit improved cell membrane integrity, increased anthocyanin biosynthesis and accumulation of osmolytes, reduced stomatal aperture, enhanced leaf water potential, decreased shoot growth and increased root growth (reduced shoot/root ratio), resulting in higher survival rates (Figure 1d) (Nguyen *et al.,* 2016; Nishiyama *et al.,* 2011, 2013). Thus, studies using CTK‐deficient Arabidopsis lines, such as quadruple *ipt1,3,5,7* mutants, or CTK signalling mutants (e.g. *ahk2/3*, *ahp2/3/5*, or *arr1/10/12*), have provided evidence that CTK metabolism and signalling components can act as negative regulators of plant drought adaptation, as the lowered CTK levels under unfavourable conditions result in reduced plant growth rates.

In contrast to what happens with *IPT* mutant lines of Arabidopsis where the chronically low CTK levels are inversely related to drought acclimation, increases of CTK, when more tightly controlled through transgenic manipulation, can show an opposite relationship. Dexamethasone spray‐controlled stimulation of the expression of a CTK biosynthetic gene (*DEX::IPT*) or the exogenous application of CTK [meta‐topolin (mT)] at the onset of stress, has resulted in a more rapid and vigorous plant recovery after drought (Prerostova *et al.,* 2018). Also, transgenic crop plants with stress/senescence/maturation‐inducible promoters driving the expression of *IPT* genes, have shown improved plant tolerance to drought, heat, and other stresses. For example, CTK up‐regulation via overexpression of *IPT* as driven by a maturation‐inducible *AtMYB32* promoter (*AtMYB32::IPT*), a stress‐inducible *SARK* promoter (*SARK::IPT*), or a senescence‐inducible *SAG12* promoter (*SAG::IPT*), all resulted in adaptive responses under drought stress (Bedada *et al.,* 2016; Joshi *et al.,* 2019; Qin *et al.,* 2011; Rivero *et al.,* 2010; Xiao *et al.,* 2017).

The growing number of *in planta* studies in Arabidopsis have shown the multifaceted nature of *IPTs* during drought stress, indicating *IPT* can be both, a positive, or a negative regulator of abiotic stress tolerance. These studies significantly advanced our understanding of the regulation of plant morphology, molecular genetics, biochemistry, and physiology by *IPTs*, and pointed the way to some remarkable features of *IPTs* that may be used to engineer crops species such as rice, maize, wheat, and sweet potato (Table 1). Supported by the improvements in genetic engineering/genome editing tools, the research on *IPTs* is now moving from discovery‐driven research towards more practical engineering and field crop‐based applications.

### Functional specificity of IPT in response to abiotic stresses

The manipulations of CTKs in plants to cope with abiotic stresses, either by CTK exogenous application or by overexpressing CTK biosynthesis/signalling components, have brought promising outcomes (Chen *et al.,* 2020; Hallmark and Rashotte, 2019; Wang *et al.,* 2020b). Many findings have emerged, linking *IPTs* with various plant molecular and physiological responses under abiotic and biotic stresses (Cortleven *et al.,* 2019; Jameson and Song, 2016). Besides the signalling function of phytohormones, their capacity to control leaf senescence, ROS detoxification, osmotic potential, and the protection of the photosynthetic apparatus, highlight *IPT*s as key molecules in cellular responses to cold, drought, salt, metal, and biotic stresses.

#### Osmotic and temperature stress regulation

##### The linkages between IPTs and hormonal crosstalk

**Figure 3:**
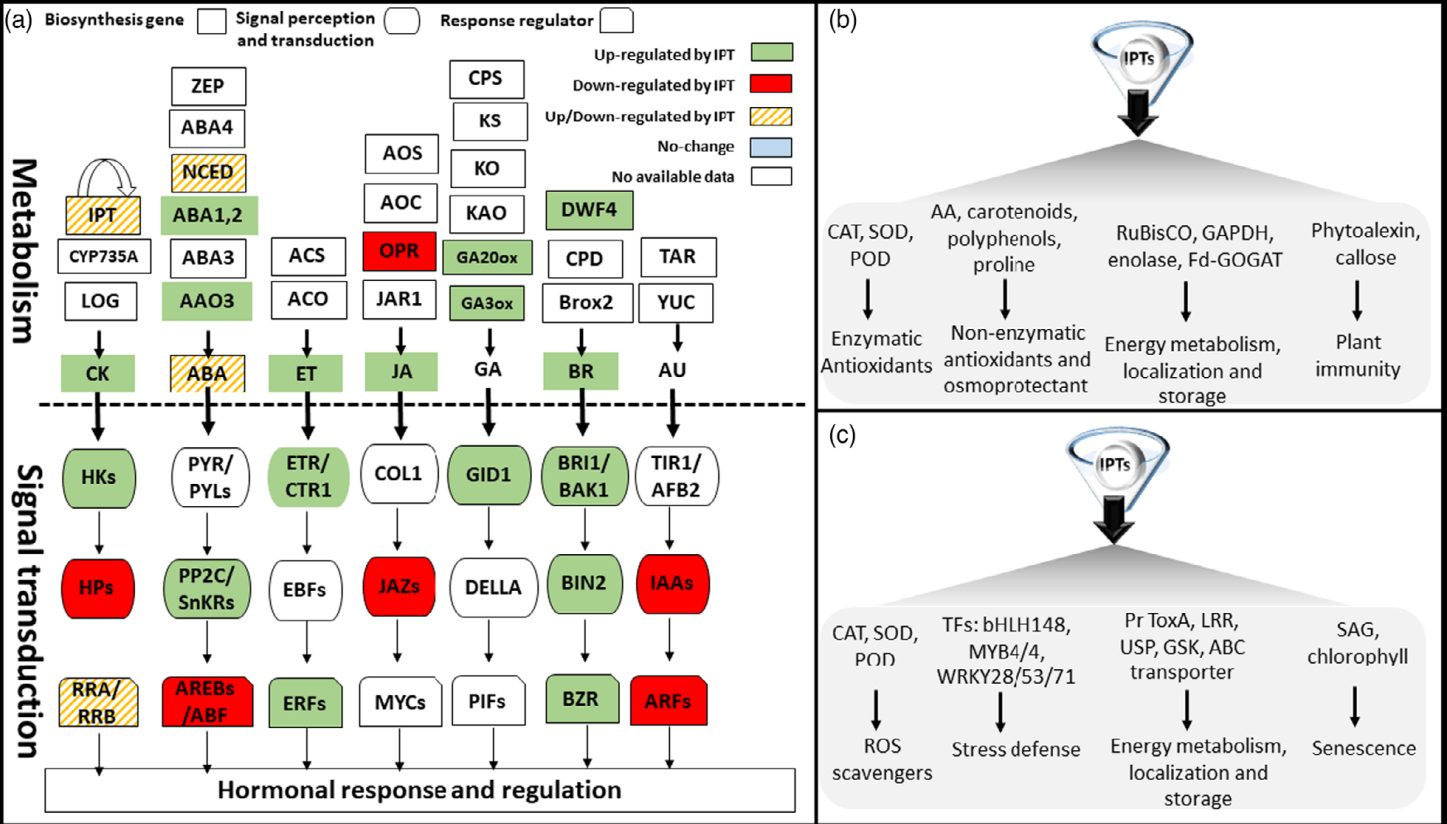
Functional specificities of *IPT* and their potential utilization for crop improvement. Manipulation of specific *IPTs* can help to obtain a certain trait, enhancing the feasibility of goal‐directed molecular design (Kuppu *et al.,* 2013; Nishiyama *et al.,* 2011; Peleg *et al.,* 2011; Rivero *et al.,* 2010; Skalák *et al.,* 2016; Xu and Huang, 2017). (a) Schematic diagram representing contribution of *IPT*s in the regulation of CTKs with other plant hormone biosynthesis and signalling pathways. The results of *in planta* studies report that overexpression of *IPTs* changes endogenous CTK and other phytohormone levels. Manipulation of specific *IPT*s modifies transcriptional activities of hormone biosynthesis and signalling components. Cytokinin: CTK; IPT: isopentenyl transferase; CYP735A: cytochrome P450 monooxygenase, family 735, subfamily A (cytokinin *trans*‐hydroxylase); LOG: cytokinin phosphoribohydrolase ‘Lonely guy’; HK: histidine kinase; HP: histidine phosphotransfer; RRA/RRB: response regulator type A/B. Abscisic acid: ABA; ZEP: zeaxanthin epoxidase; ABA1,2: zeaxanthin epoxidase ABA deficient 1/2; ABA3: molybdenum cofactor (MoCo) synthase; ABA4: unidentified enzyme; AAO3: abscisic aldehyde oxidase 3; PYR: pyrabactin resistance; PYLs: pyrabactin 1‐Like; PP2Cs: protein phosphatase 2Cs; SnKRs: sucrose non‐fermenting1‐related protein kinase2; ABREs: ABA‐responsive elements; ABF: ABRE‐binding factors. Ethylene‐ET; ACS: 1‐aminocyclopropane‐1‐carboxylic acid (ACC) synthase; ACO: ACC oxidase; ETR: ET receptor; CTR1: constitutive triple response 1; EBFs: ET‐intensive (EIN3) binding F‐box proteins; ERF: ethylene response factor. Jasmonic acid: JA; AOS: Allene Oxide Synthase; AOC: Allene Oxide Cyclase; OPR: 12‐oxo‐phytodienoic acid (OPDA) reductase; JAR1: JA resistant 1; COL: constant‐like; JAZ: jasmonate ZIM‐domain; MYCs: basic helix–loop–helix (bHLH) transcription factors. Gibberellin: GA; CPS: ent‐copalyl diphosphate synthase; KS: ent‐kaurene synthase; KO: ent‐kaurene oxidase; KAO: ent‐kaurenoic acid oxidase; GA20ox: GA 20‐oxidases; GA3ox: GA 3‐oxidases; GID1: Gibberellin insensitive dwarf1 (GA receptor); DELLA: GA signalling repressors; PIFs: phytochrome interacting factors. Brassinosteroids: BR; DWF4: Dwaft4 (a cytochrome P450); CPD: BR biosynthetic cytochrome P450; BR6ox2: BR‐6‐oxidase2; BRI1: BR‐insensitive1; BAK1: BRI1‐associated‐kinase1; BIN2: BR insensitive2; BZR: Brassinazole‐resistant1. Auxin: AU; TAR: tryptophan aminotransferase‐related; YUC: YUCCA enzyme; TIR1: Transport inhibitor response1; AFB2: Auxin signalling F‐box2; IAAs: Indole‐3‐acetic acid transcriptional repressors; ARFs: Auxin response factors. Figure design adapted from (Nishiyama *et al.,* 2018). (b) At the protein level, increasing endogenous CTK levels through the controlled expression of *IPT* GFMs can target various substrates to regulate specific functions. Arrowheads represent activation. AA: ascorbic acid; CAT: catalase, SOD: superoxide dismutase, POD: peroxidase; RuBisCO GAPDH: Ribulose‐1,5‐bisphosphate carboxylase/oxygenase; GAPDH: Glyceraldehyde‐3‐phosphate dehydrogenase; Fd‐GOGAT: Ferredoxin‐dependent glutamate synthase. (c) At the transcriptional level, *IPTs* act upstream of various genes to regulate specific functions. Arrowheads represent activation. ROS: reactive oxygen species; TFs: transcription factors; bHLH148: basic helix–loop–helix 148; MYB4/4: MYB domain protein 4; WRKY28/53/71: TFs containing highly conserved WRKY domain; Pr ToxA: proteinaceous host‐selective toxin; LRR: leucine‐rich repeat; USP: universal stress protein; GSK: glycogen synthase kinase; ABC: ATP‐binding cassette transporters; SAG: senescence‐associated gene.

Many phytohormones are involved in signal transduction networks of CTKs during plant responses to abiotic stress (Figure 3a). In Arabidopsis, *IPTs* have emerged as players in ABA‐mediated signalling pathways (Nishiyama *et al.,* 2011). Generally, *IPTs* are suppressed upon ABA treatment, and thereby collaborate with ABA in drought stress alleviation (Liu *et al.,* 2013b; Nishiyama *et al.,* 2011).

Manipulation of CTKs in plants by targeting CTK metabolic genes affects ABA content. Transcriptionally, *IPT* genes are down‐regulated by exogenous ABA treatment suggesting that stress‐induced ABA might down‐regulate CTK levels and facilitate plant adaptation to adverse environmental conditions. Results of CTK metabolism and signalling studies suggest that *IPT*s contribute to the antagonistic actions between ABA and CTKs under water deficit conditions (Huang *et al.,* 2018b; Li *et al.,* 2016). Drought exposure increases ABA content, which activates the *AtMYB2* transcriptional factor (TF) (Osakabe *et al.,* 2014). The *AtMYB2* TF subsequently down‐regulates several *IPT* genes, namely *IPT1, 4, 5, 6*, and *8*, resulting in a reduction in endogenous CTK levels (Abe *et al.,* 1997; Guo and Gan, 2011). Also consistent with an antagonistic CTK/ABA relationship, an eggplant *IPT* overexpressor had reduced levels of ABA under drought conditions which helped delay leaf senescence and induce abiotic stress tolerance (Xiao *et al.,* 2017).

Research into the sophisticated and complex mechanisms of CTK‐ABA crosstalk in response to osmotic stress has been conducted to find out how ABA and CTKs antagonistically regulate drought stress response in plants (Huang *et al.,* 2018b). In this regard, the reduction in CTK levels enables plants to cope with water deficit through a wide range of morphological and biochemical changes such as effective allocation of nutrient resources for root development, and enhanced ability to access water (Figure 1d). However, SAG12*::IPT*‐transgenic Arabidopsis had elevated ABA levels, which was opposite to ABA levels observed in *DEX::IPT* plants under drought stress (Prerostova *et al.,* 2018). Similarly, an *IPT*‐transgenic tomato using the senescence/stress‐activated promoter, *SARK*, resulted in the induction of the carotenoid pathway leading to enhanced ABA biosynthesis (Rivero *et al.,* 2010). Thus, constructs with differently‐driven promoters engineered with *IPT* genes can result in divergent profiles of hormone homeostasis and thereby induce dissimilar physiological responses in the transgenic plants (Li *et al.,* 2019).

The expression of *IPT*s is affected by stress conditions and *IPTs* directly modify the content of CTKs and affect the content of other hormones through CTK action under abiotic stress (Figure 3a). In cotton, transcriptomic analyses of *IPT* overexpressors revealed an up‐regulation of ethylene (ET), brassinosteroids (BRs), JA, auxin, gibberellin (GA), and ABA‐related genes. In rice, *SARK::IPT* plants had increased expression of BR‐related genes and repressed expression of JA‐related genes (Peleg *et al.,* 2011). In broccoli, both exogenous benzylaminopurine (BA) treatment and *SAG::IPT*‐induced elevation of CTKs resulted in reduced post‐harvest senescence via antagonist action with ET (Liu *et al.,* 2013a). Ectopic expression of *IPT* in Arabidopsis displayed higher accumulations of JA, jasmonate‐isoleucine, and an ethylene precursor (aminocyclopropane carboxylic acid (ACC)) under drought conditions (Prerostova *et al.,* 2018). Given the importance of *IPT*s in hormonal crosstalk, and their capacity to regulate various metabolic/signalling components in different phytohormone pathways (Figure 3a), there is a compelling case to undertake future research about how these modifications correspond to agronomic traits.

##### Delayed senescence

In addition to natural ageing factors, senescence can be triggered by abiotic stress. This phenomenon limits crop productivity via degradation of chloroplasts, reduced photosynthesis, massive degradation of organelles and macromolecules, and re‐allocation of nutrients (Zwack and Rashotte, 2013). Senescence is widely marked by a decline in the content of biologically active CTK forms in the leaf. As an inhibitor of senescence, CTK‐induction by *IPTs* can represent an effective strategy to reduce leaf senescence (Cortleven *et al.,* 2019). There are already many examples of genetic manipulations of *IPT* that led to beneficial phenotypes via anti‐senescence effects of CTKs. For example, transgenic *AtMYB2xs::IPT* wheat demonstrated delayed leaf senescence and higher leaf water potential under both well‐watered and water stress conditions (Joshi *et al.,* 2019). These features were also found in transgenic *IPT* maize, which had superior grain yields (Bedada *et al.,* 2016). In eggplants, delayed senescence was found in *IPT*‐transgenic plants with enhanced cold/drought tolerance (Xiao *et al.,* 2017). Overexpression of *IPT* in broccoli helped delay senescence and post‐harvest senescence (Liu *et al.,* 2013a). Over the years, several molecular studies confirmed positive functions of inducible expression of *IPTs* which specifically delay senescence and thereby improve yield under drought stress conditions. This was achieved in petunia (Chang *et al.,* 2003), tomato (Luo *et al.,* 2005), cauliflower (Nguyen *et al.,* 2008), creeping bentgrass (Merewitz *et al.,* 2010), cotton (Kuppu *et al.,* 2013), and canola (Kant *et al.,* 2015). These results are critically important as they indicate that *IPTs* can be key targets for the development of transgenic crops with an enhanced ability to grow under reduced irrigation and without incurring yield penalties, ultimately contributing to savings in irrigation water. In general, active CTK levels decrease during leaf senescence. However, *N7*‐glucosides and *O‐*glucosides (in particular *t*ZOG) are known to accumulate in senescing leaves (Šmehilová *et al.,* 2016). Exogenously applied *t*Z and its glucoside derivatives (*t*ZNGs: *t*Z7G, *t*Z9G) both delayed senescence in Arabidopsis and *t*ZNGs altered plant transcriptome and proteome distinctly from the changes caused by *t*Z (Hallmark *et al.,* 2020). A biological role in delaying leaf senescence through activation of CTK‐associated genes has been observed also for iP and its glucoside isopentenyladenine‐9‐glucoside (iP9G) (Hallmark and Rashotte, 2020). Manipulation of CTKs to increase plant yield either by targeting CTK metabolic genes or through exogenous hormone applications has provided promising outcomes (Gu *et al.,* 2015; Holubová *et al.,* 2018; Wang *et al.,* 2020b; Zhao *et al.,* 2015). The observed yield increases have been attributed mainly to delayed senescence and not to direct impact on mitotic cell division during endosperm development. However, this distinction remains quite uncertain and more effort is required to generate detailed data sets to complete a full inventory of *IPTs* involved in endosperm development (coenocyte, cellularization, cell division, expansion, differentiation, and maturation) at molecular, cellular, and tissue levels. For example, transcriptome analysis revealed the importance of CTK signalling during early endosperm development in Arabidopsis (Day *et al.,* 2008).

##### Modification of major metabolic events and ROS detoxification pathways

Cellular events, such as alterations in carbohydrate and amino acid fluxes, are fundamental features in plant capacity to successfully cope with major osmotic stresses and involve a redirection of resources away from growth pathways and towards stress‐defensive responses. These events can be coordinated by *IPTs*, which induce major changes in active CTKs and cellular metabolism to affect the whole plant body which, in turn, induces stress acclimation/adaptation responses. For example, *IPT* overexpressing plants tolerate drought by maintaining normal accumulations of amino acids, sugars, and organic acids involved in the citric acid cycle (Merewitz *et al.,* 2012). Also, overexpression of *IPT* regulates sink strength and coordinates regulation of carbon and nitrogen assimilation in rice under drought stress (Peleg *et al.,* 2011; Reguera *et al.,* 2013). In broccoli, SAG::*IPT* transgenes moderately increased levels of carbohydrate metabolism proteins and more strongly increased stress defence proteins, known as molecular chaperones (Liu *et al.,* 2013a). Likewise, work with broccoli demonstrated that increasing CTK levels, either by overexpressing an *IPT* gene or exogenous hormone treatment, can regulate genes involved in sugar transport, and the metabolism of carbohydrates, amino acids, and lipids (Gapper *et al.,* 2005; Liu *et al.,* 2013a).

Reactive oxygen species (ROS) have partially reduced or excited states of molecular oxygen, and they are the unavoidable toxic by‐products of aerobic metabolism that have accompanied aerobic life forms since about 2.4–3.8 billion years ago (Mittler, 2017). Abiotic stress leads to excessive accumulation of ROS causing oxidative stress, leading to protein denaturation, lipid peroxidation, and nucleotide degradation. Eventually, this results in cellular damage and ultimately cell death (Choudhury *et al.,* 2017). At the cellular level, ROS can be scavenged via non‐enzymatic systems (ascorbic acid, glutathione, tocopherols, carotenoids, phenols, etc.), enzymatic systems (CAT, SOD, POD, APX, etc.), and the osmolyte, proline (Das and Roychoudhury, 2014). *IPT*s were found to activate acclimation responses, as a result of alterations in the redox state of regulatory proteins, by way of transcription and translation, thereby mitigating effects of stress on metabolism and reducing metabolic ROS levels (Figure 3b) (Lai *et al.,* 2007; Merewitz *et al.,* 2012; Skalák *et al.,* 2016; Thomas *et al.,* 1995; Xu *et al.,* 2016). For example, overexpressing *IPT,* under the control of SAG12, that was correlated with the elevation of the antioxidant enzyme activities, promoted cotton seed germination and seedling tolerance to salt stress (Liu *et al.,* 2012; Shan *et al.,* 2019). A similar construct in eggplant enhanced plant cold/drought tolerance and stimulated higher activities of ROS‐scavenging enzymes (Xiao *et al.,* 2017). Enhancing CTK synthesis by overexpressing *SAG12*::*IPT* alleviated drought‐related inhibition of root growth and activated ROS‐scavenging systems in creeping bentgrass (Xu *et al.,* 2016).

##### Protection of photosynthetic machinery

Photosynthesis is one of the most susceptible cellular processes that strongly respond to the effects of abiotic stress (Gururani *et al.,* 2015). *IPT*‐induced CTKs, with their clear impact on the protection of the photosynthetic apparatus, can reduce the penalty on photosynthetic rate caused by drought stress, via regulation of stomatal conductance (Rivero *et al.,* 2007) and chlorophyll biosynthesis (Xiao *et al.,* 2017; Zhang *et al.,* 2010). Transgenic peanut (*SARK::IPT*) was observed to have higher photosynthetic rates, stomatal conductance, transpiration, and yield under drought stress (Qin *et al.,* 2011). Similarly, the enhanced CTK synthesis in tobacco expressing *SARK::IPT* prevented the degradation of photosynthetic protein complexes during drought (Rivero *et al.,* 2010). Overexpression of *IPT* in canola was associated with higher chlorophyll levels, delay in leaf senescence, and enhanced yield under rainfed and irrigated conditions (Kant *et al.,* 2015). To elucidate the effects of *IPT*‐altered CTKs on the proteome of the chloroplast and its subfractions (stroma and thylakoids), transgenic *pSSU::IPT* tobacco plants, that had high levels of CTKs, were analysed. Results revealed substantial quantitative differences in stroma proteins with significantly increased levels of CTKs in the transgenic plants but with no qualitative changes in the chloroplast proteomes between the transgenic and wild‐type plants (Cortleven *et al.,* 2011). Paradoxically, such excessive amounts of CTKs do not result in any significant improvement in the chloroplast proteomes, and this emphasizes the important task of targeting the right hormone balance when employing any overexpression of *IPTs* for crop improvement. Overall, genetic engineering of *IPTs* in crops can ameliorate stress impacts and promote photosynthesis at different physiological and cellular organization levels under abiotic stress (stay‐green, leaf development, plastid function, protection of photosynthetic proteins, influence on photosynthetic genes, etc.) (Kant *et al.,* 2015; Rivero *et al.,* 2010).

##### Modulation of transcriptomic and proteomic responses

*IPT* is relevant to an array of abiotic stress responses, and its precise induction is associated with modified gene expression. Developments in plant biotechnology (the advances of transcriptomic approaches) flanked by parallel progress of *in planta* studies have enabled the elucidation of downstream transcriptional regulation of the *IPTs* involved in abiotic stress responses in plant genetic models and crop species. These diverse mechanisms include energy production, metabolic activities, stress defence, signalling, protein synthesis and transport, and membrane transport (Figure 3c).

In drought‐stressed creeping bentgrass, many downstream communicators were exposed by the use of a transgenically enhanced *IPT* (*SAG12::IPT)* and these included an oxygen‐evolving enhancer protein 3–1 and chloroplast precursor (OEE3) (metabolism), RuBisCo large subunit (energy production), leucine‐rich repeat (LRR) receptor‐like kinase (transmembrane receptor proteins), universal stress protein 5327 (stress defence) and CAT (ROS detoxification) (Merewitz *et al.,* 2016). The analysis of differentially expressed genes (DEGs) in the transgenic *IPT* creeping bentgrass found that 170/250 DEGs related to energy production, metabolism, stress defence, signalling, protein synthesis and transport, and membrane transport under drought (Merewitz *et al.,* 2016). Plant adaptive responses to abiotic stress are frequently activated by TFs, which are involved in the repression/activation of stress‐responsive genes (Colinas and Goossens, 2018; Ohama *et al.,* 2017). Among 127 differentially expressing TFs in *IPT*‐transgenic creeping bentgrass, 65 exhibited up‐regulation and 62 were down‐regulated as compared to non‐transgenic plants. These up‐regulated gene expressions were involved in central hubs of *bHLH148*, *MYB4/4*‐like, and *WRKY28/53/71* genes, which are associated with JA signalling and with down‐regulated, ABA signalling‐associated genes (Xu and Huang, 2017). By contrast, mutant Arabidopsis (quadruple *ipt1,3,5,7* loss of function) displayed an up‐regulation of ABA marker genes (*AIL1, COR47, RAB18, RD29B, SAG29*), some TFs (*NAC*, *DREB,* and *ZFHD*), and several functional proteins that improved osmotic stress tolerance (Nishiyama *et al.,* 2011, 2012).

Because *IPTs* regulate gene expression in many cellular metabolic pathways (Figure 3), regulation is also manifested at the level of proteomic responses under abiotic stress. Proteome analyses of transgenic plants (*SAG12::IPT* and *HSP18::IPT*) have revealed relatively high accumulation rates of leaf proteins (enolase, oxygen‐evolving enhancer protein 2, putative oxygen‐evolving complex, Rubisco small subunit, Hsp90, and glycolate oxidase) and root proteins (Fd‐GOGAT, nucleotide‐sugar dehydratase, NAD‐dependent isocitrate dehydrogenase, ferredoxin‐NADP reductase precursor, putative heterogeneous nuclear ribonucleoprotein A2, ascorbate peroxidase, dDTP‐glucose 4–6‐dehydratases‐like protein) as compared to non‐transgenic lines when exposed to heat stress (Xu *et al.,* 2010). Drought tolerant *SAG12‐IPT* creeping bentgrass displayed an abundance of proteins involved in: photosynthesis and respiration [ribulose 1,5‐bisphosphate carboxylase (RuBisCO) and glyceraldehyde phosphate dehydrogenase (GAPDH)]; amino acid synthesis (methionine and glutamine); protein synthesis and destination [chloroplastic elongation factor (EF‐Tu) and protein disulphide isomerases (PDIs)] (Figure 3b) (Merewitz *et al.,* 2011). These results indicate that *IPT*‐induced endogenous CTKs may directly and/or indirectly regulate functional proteins involved in the above‐mentioned cellular pathways, thereby reducing stress penalties. Researchers are getting closer to completing a full inventory of downstream regulatory mechanisms of *IPTs* which are involved in plant stress adaptation/acclimation, but they are still far from completing a comprehensive view of the signalling cascades that operate together to achieve final outputs at the molecular, cellular, and tissue level as well as long‐distance signalling under stress (e.g. CLAVATA3/EMBRYO‐SURROUNDING REGION‐RELATED 25 (CLE25) peptide) (Takahashi *et al.,* 2018).

#### Metal stress response

Isopentenyltransferases are of particular importance for plant metal stress responses as their overexpression can result in improved heavy metal stress tolerance (Gomez Mansur *et al.,* 2021; Thomas *et al.,* 2005). Accordingly, *IPT*‐induced CTKs in transgenic tobacco enhanced copper stress tolerance, and this was explained by an increased expression of a metallothionein‐like gene (Thomas *et al.,* 2005). Likewise, a transgenic wheat line, overexpressing *IPT* under the *SARK::IPT* promoter, showed a lower reduction in root growth under cadmium stress, and this was attributed to the activation of phenolic secondary metabolism, increased antioxidant defences, and cell wall reinforcement (Gomez Mansur *et al.,* 2021). Another study, using Arabidopsis, reported an opposite relationship whereby, CTK depletion plants (*ipt1,3,5,7* loss of function) gained selenium tolerance through the induced antioxidant enzyme activities and increased glutathione (GSH) content (Jiang *et al.,* 2019). However, the antibody method used for CTK profiling was limited in its ability to discriminate among different CTK types and their identification and quantification should be interpreted with appropriate caution.

### Biotic stress response

Along with SA and JA, CTKs are involved in plant responses to biotic stress factors (Akhtar *et al.,* 2020; Ciura and Kruk, 2018). Therefore, manipulating genetic elements involved in CTK metabolism has potential to augment plant performance under biotic stress. *In planta*, overexpression of *IPT* genes has been demonstrated to promote plant immunity against bacteria, fungi, and insects (Choi *et al.,* 2010; Großkinsky *et al.,* 2011; Smigocki *et al.,* 2000).

#### IPTs in bacterial/fungal pathogen and insect tolerance

While *IPT* activity and CTK accumulation are effective means by which plants deal with abiotic stresses they are also strong effectors for biotic stress tolerance. Increases in endogenous CTK level in tobacco, using a binary *pOp‐ipt/LhGR* system for dexamethasone‐inducible *IPT* expression (*DEX::IPT*), triggered rapid necrotic lesions on leaves that could act as a response to the plant detection of an ‘intruder’ attack, and limit the rate of the pathogen expansion (Novák *et al.,* 2013). By overexpressing a bacterial *IPT* driven by a pathogen‐inducible synthetic promoter (*4xJERE*; 4x jasmonate and elicitor‐responsive expression), *SAG12* (senescence‐associated gene 12), or *TET* (tetracycline‐dependent) promoter, transgenic tobacco was rendered significantly less susceptible to *Pseudomonas syringae* pv *tabaci* (*Pst*) while maintaining only mild symptoms of wildfire disease at infected sites (Großkinsky *et al.,* 2011). This feature helps prevent the spread of bacteria as well as decreases the enlargement of the necrotic lesions. At the molecular level, *IPT* contributed to bactericidal activity of the transgenic tobacco through the expression of *EAS* and *C4H*, which encode for two antimicrobial phytoalexin compounds, scopoletin, and casidiol, respectively (Großkinsky *et al.,* 2011). Individually overexpressing *AtIPT1*, *3*, *5*, or *7*, driven by the *35S* promoter, mitigated the damage caused by *Pseudomonas syringae* pv. *tomato* DC3000 (Pst DC3000) in Arabidopsis by reducing pathogen growth (Choi *et al.,* 2010). A *35S::IPT3* transgenic Arabidopsis displayed significantly stimulated callose deposition when treated with Pst DC3000 while there was no callose accumulation observed in wild‐type plants (Choi *et al.,* 2010). Callose deposition is one of the primary defence responses that relates to plant cell wall reinforcement against pathogen attack, and it is often used as a parameter to evaluate plant immunity (Fan *et al.,* 2020; Liu *et al.,* 2020). Besides suppressing Pst DC3000 invasion, transgenic *35S::IPT3* Arabidopsis had improved resistance against a virulent necrotrophic fungus, *Alternaria brassicicola* (Choi *et al.,* 2010). Reusche *et al*. (2013) showed that transgenic Arabidopsis overexpressing bacterial *IPT* under the regulation of the *SAG12* promoter resulted in fewer chlorotic and necrotic leaves and less stunted growth compared with wild‐type plants upon exposure to infection by the fungus *Verticillium longisporum*. Additionally, *V. longisporum*‐infected Arabidopsis showed significant increases in expression of *CKX1, CKX2, and CKX3*, and this was consistent with a decrease in *t*Z level observed during fungal infection (Reusche *et al.,* 2013). Transgenic *IPT* counteracted the CTK degradation normally prompted by infection of *V. longisporum*, producing an antifungal phenotype in host Arabidopsis.

Our understanding of the role of *IPT* genes in response to insect attack is quite limited compared with studies of pathogenic microbe infections and the few known examples suggest the existence of insect‐host plant‐specific mechanisms that regulate *IPT* involvement in plant defence reactions. Smigocki *et al*. (1993, 2000) had investigated an association between elevated CTK level and enhanced insecticidal effect in three transgenic plants that all carried *PI‐II* *(Proteinase inhibitor‐II)‐IPT* gene construct: *Nicotiana plumbaginifolia*, *Nicotiana tabacum,* and *Lycopersicon esculentum* (tomato). Both transgenic *N. plumbaginifolia* and transgenic tobacco exhibited robust tolerance against *Manduca sexta* with 50% to 70% less leaf consumption (Smigocki *et al.,* 1993, 2000). Leaf extracts of transgenic *N. plumbaginifolia* had greater lethality to *M. sexta* second instar larvae, compared with less active suspension of the transgenic tobacco leaf (Smigocki *et al.,* 2000) while anti‐insect effect on *M. sexta* was less consistent in the transgenic tomato since the reduction in larval weight gain could not be repeated in two independent experiments (Smigocki *et al.,* 2000). On the other hand, analysis of the feeding habits of another insect herbivore, *Tupiocoris notatus*, indicated that the feeding damage on tobacco was more profound on the leaves with increased CTK levels (Brütting *et al.,* 2018). Overall, the available data suggest that altering *IPT* gene expression can be used to modulate plant tolerance to insect attacks.

#### IPT involvement in crosstalk between CTKs and other hormones during biotic stress responses

Salicylic acid and JA are central factors in plant immune systems (Ávila *et al.,* 2019; Zhang and Li, 2019). Previous study has elucidated the crosstalk between CTKs and SA signalling components in Arabidopsis via interactions between a type B ARR CTK transcription factor (*ARR2*) and the SA response factor (*TGA3*), the latter of which is required to induce the expression of SA‐related genes including defence marker gene *PR1* (pathogenesis related 1) (Choi *et al.,* 2010). Elevated endogenous CTK levels in transgenic *35S:IPT3 Arabidopsis* plants significantly induced the expression of a *PR1* gene upon the infection of *P*. *syringae* pv. *tomato* DC3000 (Choi *et al.,* 2010), probably via the promotion of the CTK‐dependent phosphorylation of *ARR2*. By contrast, in tobacco, CTK‐induced immunity was reported to be SA‐independent, since there was no significant change in SA levels and only slight changes in transcription levels of SA signalling components, *NPR1* and *PR1,* which mediate resistance against *P*. *syringae* (Großkinsky *et al.,* 2011). Similarly, transgenic *4xJERE:IPT* tobacco did not show any enhancement of JA levels, regardless of exposure to pathogenic infection (Großkinsky *et al.,* 2011). In cotyledons of common bean, the accumulation of bactericidal phytoalexins was induced by exogenous SA (Durango *et al.,* 2013) while in *IPT*‐transgenic tobacco, phytoalexin production was stimulated by elevated CTK levels, with SA levels remaining unaffected (Großkinsky *et al.,* 2011). These observations suggest that synthesis of phytoalexins could be driven separately by *IPT*‐induced CTKs or by CTKs in concert with SA (Jeandet *et al.,* 2013). However, more research is still needed in plant anti‐pathogen responses to clarify the involvement of *IPT* genes in interactions among CTKs and immune hormones, including SA and JA, as well as other hormones including auxin and ABA (Huang *et al.,* 2018a; Shen *et al.,* 2018), especially in important crop species such as rice, maize, wheat, or soybean.

## Timing and design of IPT manipulations determine the extent of crop productivity and sustainability

Cytokinins exert many of their phenotypic effects by changing the nutrient source‐sink relationships among organs like seeds, pods, stems, leaves, and roots (Roitsch and Ehneß, 2000; Werner *et al.,* 2008). The very nature of this dynamic means that spatial and temporal control of CTK production must be strategically and tightly controlled. Indeed, many early attempts at *IPT* transformation of crops for improved yields were thwarted by poorly controlled *IPT* expression with constitutive or leaky promoters (Atkins *et al.,* 2011; Jameson and Song, 2016). This was often accompanied by systemic increases in CTKs, and off‐target growth changes such as hyperbranching, inhibited root production, and delayed senescence (Kuppu *et al.,* 2013; Nawiri *et al.,* 2018; Xiao *et al.,* 2017). Even if expression is successfully localized within yield‐defining organs (i.e. in the flower or seed), excess CTKs can enter the vasculature and be translocated far from the point of synthesis (Atkins *et al.,* 2011). Thus, the design of constructs and choice of promoter moieties will define if the *IPT* expression will be correctly targeted at the right tissue or organ and the right moment in development. In this regard, previously established *IPT*‐transgenic plants with strong constitutive promoters (*35S* promoter) and inducible heat shock‐responsive promoters (*Phsp70* promoter), resulted in abnormally high endogenous CTK levels and exhibited the retardation phenotype (Loven *et al,* 1993; Smigocki and Owens, 1988). More narrowly responsive promoter constructs such as the senescence‐specific promoter, *SAG12*, a stress‐ and maturation‐induced promoter from the senescence‐associated receptor protein kinase (*SARK*) gene, and a developmental process‐related promoter from the *AtMYB32* gene have been used to express *IPT* in a more controlled manner (Table 1). These have all been met with varying levels of success in improving seed yields, increasing canopy cover and shoot biomass, and improved water relations and drought tolerance (Chang *et al.,* 2003; Gan and Amasino, 1995; Joshi *et al.,* 2019; Kant *et al.,* 2015). However, the success has not been absolute, and complications usually accompany the transgenic performance including morphological and physiological abnormalities such as delayed flowering, and nutrient deficiencies to name a few (Cowan *et al.,* 2005; Jordi *et al.,* 2000; McCabe *et al.,* 2001). It is clear that more work on the strategic control of *IPT* expression needs to be done to refine CTK production with more precision.

One of the most potentially confounding issues of spatial *IPT* gene expression is the fact that CTKs can cause very different source‐sink effects between roots and shoots. In fact, not only does expression of various *IPT*s occur in different parts of the plant body, but also biosynthesis of different CTK forms is spatially diverse among plant organs (Durán‐Medina *et al.,* 2017; Kiba *et al.,* 2019). For example, *t*Z biosynthesis is mainly localized in the root tissues, while iP‐type CTKs are produced both in roots and in aerial plant parts (Ko *et al.,* 2014). As long‐distance messengers, CTK ribosides are transported through the plant vascular system to their sites of action (Glanz‐Idan *et al.,* 2020; Osugi *et al.,* 2017). Cytokinins have contrasting effects on root and shoot development – they stimulate shoot growth, photosynthesis rates, and biomass accumulation; but they inhibit primary and lateral root elongation and branching (Ko *et al.,* 2014; Ramireddy *et al.,* 2018; Stenlid, 1982; Werner *et al.,* 2010). Cytokinins regulate development of root vasculature, nodule formation, nutrient uptake, and allocation; however, stimulating CTK biosynthesis in the roots may alter inter‐organ source‐sink relationships and negatively affect shoot traits that contribute to yield. This emphasizes that manipulation of *IPT* expression must be spatially and tightly controlled to balance root and shoot phenotypes and to stabilize, or even enhance, crop yields.

Reprogramming endogenous CTK profiles for modification of plant architecture to improve resistance to stress can be achieved by two approaches. The first is by decreasing CTK content in roots by overexpression of *CKX* genes, which confers enhanced root fitness and remarkable phenotypic plasticity (Macková *et al.,* 2013; Pospíšilová *et al.,* 2016; Ramireddy *et al.,* 2018; Werner *et al.,* 2010). The second approach is realized by increasing endogenous CTK content via inducible expression of *IPT* genes to improve plant acclimatization/adaptation, or to delay senescence and minimize yield losses. This indirect mechanism could be used to limit damage caused by stress, by engineering stress‐ or senescence‐induced expression of *IPT* genes with specific promoters like the maturation‐inducible *AtMYB32*, the stress‐inducible *SARK*, or a senescence‐inducible *SAG12*, or a dexamethasone‐inducible *pOp/LhGR* (Table 1). Importantly, whenever CTK levels are elevated, via ectopic *IPT* expression or exogenous CTK treatment, increased transcriptional levels of *CKX* genes and/or CKX activity occurs (Panda *et al.,* 2018; Prerostova *et al.,* 2018). Positive correlations in gene expression of *IPT* and *CKX* GFMs were found in maize kernels (Brugière *et al.,* 2008), rapid cycling field mustard (O’Keefe *et al.,* 2011), wheat seed development (Nguyen *et al.,* 2020; Song *et al.,* 2012) and forage brassica (Song *et al.,* 2015). Regulation of *IPT* and/or *CKX* genes in relationship to CTK metabolism and plant acclimation/adaptation might involve different stress tolerance pathways and crosstalk with other phytohormones (Figure 3). For example, CTKs regulate auxin‐efflux and influx carriers (Šimášková *et al.,* 2015; Street *et al.,* 2016) to control aspects of root development, such as root formation, emergence, elongation, and gravitropism (Inahashi *et al.,* 2018). Understanding of these mechanisms and strategic promoter design can establish a scheme for the development of drought‐tolerant and high‐yielding crops through preprogrammed, via *IPT*, endogenous CTK levels.

The contribution of CTKs to crop yield determination has been thoroughly reviewed elsewhere (Chen *et al.,* 2020; Jameson and Song, 2016). There are a number of studies linking seed yield in rice, soybean, barley and wheat with the increased CTK levels, and particularly with the higher levels of *IPT* expression/activities with various genetic modulation designs (Jameson and Song, 2016; Kambhampati *et al.,* 2017; Powell *et al.,* 2013). Regarding the specific function of *IPTs* in plant yield, one needs to focus on several research approaches implemented towards understanding *IPTs* and their role in controlling grain yield and enhancing crop production (Table 1).

In rice, *IPT*‐induced CTK synthesis maintained nitrogen (N) acquisition and reduced the environmental stress penalties on photosynthesis and yield (Reguera *et al.,* 2013). Panda *et al*. (2018) suggested that overexpression of rice Os*IPT9* can increase CTK level of the developing caryopses, leading to the enhanced grain filling in rice cultivars bearing large panicles with numerous spikelets, which subsequently improves yield. Many efforts have been undertaken to modify spatiotemporal expression of *IPT*s by strategically employing differently‐driven promoters to increase crop yield (Table 1). Transgenic wheat (*IPT* driven by promoter *AtMYB2xs*) had increased yield in well‐watered and water stress conditions (Joshi *et al.,* 2019). In both glasshouse and field conditions, *IPT*‐transgenic maize (*IPT* driven by the *SARK* promoter) had higher grain yield (Bedada *et al.,* 2016). Regulation of *IPT* via the *AtMYB32* promoter improved yield under rainfed and irrigated conditions in canola (Kant *et al.,* 2015). Transgenic peanut demonstrated higher photosynthetic rates and yield‐relevant traits (Qin *et al.,* 2011). Interestingly, the timing of water deficit stress was critical for *IPT*‐transgenic cotton to enable its yield advantage over control plants. If water deficit was applied before flowering, the yield of *IPT*‐transgenic cotton was higher than non‐transgenic plants; however, when water stress was at, or after, flowering, there was no difference (Zhu *et al.,* 2018). Overall, if their expression can be adequately controlled, both spatially and temporally, *IPTs* can be a key driver for seed yield, when considering multiple species that have shown improved productivity/yield under drought conditions, including: rice (Peleg *et al.,* 2011), peanut (Qin *et al.,* 2011), cotton (Kuppu *et al.,* 2013), canola (Kant *et al.,* 2015), tropical maize (Bedada *et al.,* 2016), sweet potato (Nawiri *et al.,* 2018), or wheat (Joshi *et al.,* 2019).

## Concluding remarks and future steps

A strong and constantly growing body of evidence highlights that *IPTs* play crucial roles in phytohormone crosstalk and stress‐responsive signalling pathways (Figures 1, 2, 3). These findings provide vital insights for crop breeding, especially for improving yield by increasing abiotic stress tolerance (Table 1). *IPTs* induce transcriptomic, proteomic, and metabolic responses, as well as physiological responses, enabling a more precise monitoring of, and acclimation to, abiotic stresses. We conclude that *IPTs* should be considered as master regulators of plant yield. As explained in this review, the mechanisms enhancing stress tolerance by the up‐ or down‐regulation of endogenous CTK levels might involve different pathways and crosstalk with other phytohormones (Figure 3). Seeking crops with an optimal balance of phytohormone homeostasis and/or precise responses to stress could be achieved by root‐specific or stress‐inducible promoters (Table 1).

Less clear is the contribution of *IPT* to the molecular response to metal and biotic stress resistance, as only a few cases involving CTK enhancement via *IPT*s have been shown to benefit the plant. Similarly, future research should be expanded to examine how *IPT*s are associated with signalling for other abiotic stresses and phytohormone pathways, such as metal and submergence stress, and strigolactone signalling.

Although current knowledge indicates that several *IPT*‐related genetic signalling components are required for nutrient allocation and transport, the mechanisms regarding *IPT*‐induced alterations of vascular cell differentiation relating to root/shoot fitness or the inter‐organ communication networks remain to be discovered. Further research on cell developmental and transcriptional trajectories using novel cellular imaging and single‐cell RNA sequencing techniques would enhance our understanding of these mechanisms.

Plants co‐exist with microorganisms in nature, and plant growth‐promoting microbiomes help plants resist stress via CTKs (Egamberdieva *et al.,* 2017; Goh *et al.,* 2019; Jorge *et al.,* 2019). As CTKs are interkingdom signalling molecules, investigations into the possible function of *IPTs*, and how bacteria and fungi enhance plant stress resistance would increase our understanding of these beneficial interactions and could also provide novel technologies for crop stress management.

## Conflict of interest

All authors agree to authorship and submission of the manuscript for peer review. The authors report no commercial or proprietary interest in any product or concept discussed in this article.

## Author contributions

Hai Ngoc Nguyen involved in conceptualization, visualization, writing – original draft, and writing – review and editing. Nhan Lai Dinh contributed to writing – original draft; writing – review and editing. Anna Kisiala involved in writing – review and editing. R.J. Neil Emery contributed to funding acquisition, supervision, and writing – review and editing.
